# The Utility of a Comprehensive Hip Fracture Program: Readmission Rates May Not Be a Reasonable Marker of Success

**DOI:** 10.7759/cureus.66886

**Published:** 2024-08-14

**Authors:** Sahir S Jabbouri, Ally A Yang, Peter Joo, Ali Elaydi, Anne Moore, Matthew D Riedel, Jenna Bernstein

**Affiliations:** 1 Orthopaedics and Rehabilitation, Yale School of Medicine, New Haven, USA; 2 Orthopaedics and Rehabilitation, Yale New Haven Hospital, New Haven, USA; 3 Orthopaedics, Connecticut Orthopaedics, Trumbull, USA

**Keywords:** fragility hip fractures, mortality, length of stay, readmission rates, geriatric, hip fracture program

## Abstract

Introduction

This study evaluates the effectiveness of a comprehensive hip fracture protocol, with a focus on specific readmission reasons.

Methods

A retrospective cohort study of hip fracture patients aged 60 and older who underwent surgery before (control) and after (intervention) implementation of a comprehensive hip fracture program. Objectives included identifying readmission reasons and rates, time to operating room (TOR), length of stay (LOS), reoperation, and mortality rates. Logistic regression was utilized to determine significance.

Results

One hundred and sixty-three patients (control) vs. 238 patients (intervention) were identified. The intervention group had higher odds of 90-day readmission for a medical reason (OR = 1.735, p = 0.028). Thirty-three out of forty-two patients (79%) in the control group and 68/78 patients (87%) in the intervention group were readmitted secondary to a medical reason (pulmonary etiology being the most common). Surgical-related readmissions (surgical site infections and dislocations are most common) were lower in the intervention group compared with the control group, with 10/78 patients (13%) and 9/42 patients (21%), respectively. Twenty-four-hour TOR was achieved in 125 patients (52.5%) in the intervention group vs. 70 patients (42.9%) in the control group. LOS was shorter by 1.1 days for the intervention group (p = 0.010). Mortality was lower in the intervention group.

Discussion

A comprehensive hip fracture protocol can reduce LOS, TOR, mortality rate, and even surgical-related readmissions. Readmission rates are mainly due to medical problems, which may be unavoidable and thus may not be an adequate hip fracture effectiveness metric. Potential areas of improvement and additional study may include closer internal medicine oversight and primary care follow-up after discharge.

## Introduction

A fragility hip fracture is a common reason for hospital admission in the elderly and negatively impacts quality of life [1,2]. Patients’ functional capacity deteriorates significantly, and there is up to an eight-fold increase in all-cause mortality in elderly patients after a hip fracture [2,3]. Delay in surgery combined with a lack of comprehensive and standardized care may lead to an increased risk of complications and contribute to patient morbidity and mortality [1,4,5]. Over the past two decades, various hip fracture programs have been implemented to combat these issues and reduce in-hospital complications, disability, and mortality [5]. Organized hip fracture programs have been shown to benefit not only patients but also care providers and health systems [6]. A combination of different methods, including co-management by medical physicians and/or geriatricians with orthopedic surgeons, early surgery, and early mobility, has been demonstrated to be most effective in improving the outcomes of geriatric hip fracture patients [5,6]. However, hip fracture patients are often medically complex, and readmissions can be difficult to prevent. In a retrospective review of hip fracture patients, Erlichman et al. found that having a prior admission within one year was significantly associated with a 90-day readmission after surgery [7]. Thus, hospitals may often be penalized for unpreventable readmissions in medically complex hip fracture patients.

Our Integrated Fragility Hip Fracture Program (IFHFP) was introduced on February 1st, 2016, to improve the acute medical and surgical care of hip fracture patients. It was developed by a multidisciplinary group that involved surgeons and advanced practice providers from orthopedics; physicians from internal medicine, hospitalists, geriatrics, emergency medicine, and anesthesia; and representatives from rehabilitation services, nursing, care management, pharmacy, and performance improvement [8]. Before its implementation, there was no set institutional protocol for the management of hip fractures or the time to the operating room. Our hip fracture program comprehensively standardizes preoperative, intraoperative, and postoperative care for elderly patients with hip fractures.

The purpose of this study was to evaluate the effectiveness of our IFHFP and explore specific reasons for patient readmission in patients who underwent surgery for a fragility hip fracture either before or after the implementation of the hip fracture protocol. We hypothesized that our comprehensive hip fracture program would be associated with reduced length of stay (LOS), faster time to the operating room (TOR), a lower reoperation rate, and mortality, but would unlikely impact the overall readmission rate as many of the reasons for readmission are likely due to nonmodifiable risk factors in medically complex patients.

## Materials and methods

Patient selection

This retrospective cohort study was performed at a single tertiary academic institution on patients 60 years of age and older with fragility hip fractures who underwent surgery from January 2014 to January 2016 (control group) and from February 2016 to February 2018 (intervention group) after protocol implementation on February 1st, 2016. A fragility hip fracture was defined as a hip fracture (femoral neck, intertrochanteric, or subtrochanteric) that occurred due to a low trauma incident, such as a fall from a standing height. Patients were excluded if they had high-energy trauma causing hip fractures or periprosthetic fractures and if they did not undergo surgical treatment for their fragility hip fractures.

Integrated fragility hip fracture program (IFHFP)

The pre-operative protocol included pain control with oral and IV medications as well as femoral nerve blocks, medical clearance from a dedicated hip-fracture medicine service, subcutaneous heparin for pre-operative venous thromboembolism (VTE prophylaxis), surgical site infection prevention, and tranexamic acid (TXA). Surgical site infection prevention included hair clipping before chlorhexidine gluconate, patient skin decolonization the night before and the morning of surgery, pre-op nasal povidone-iodine, and preoperative antibiotics. Unless medically cleared, the goal of surgery within 24-36 hours after admission was set.

A dedicated hip fracture OR was assigned daily with a hip fracture on-call surgeon from a shared community and faculty surgeon call schedule. Treatment with open reduction internal fixation (ORIF), hemiarthroplasty, or total hip arthroplasty was ultimately at the discretion of the surgeon and patient preference. Standard intraoperative care included TXA before incision, a five-minute surgical site scrub, sterile site preparation with chloraprep or duraprep, normothermia of >36°C, dilute betadine lavage, and anti-bacterial occlusive dressing. 1 gram of vancomycin powder was added based on the surgeon's preference.

The postoperative protocol included DVT prophylaxis for 35 days, scheduled delirium confusion assessments, oral care, nutrition care, early mobility, and daily physical and occupational therapy starting the day after surgery. Urinary catheters placed in the operating room were removed immediately post-procedure, either intraoperatively or in the PACU.

Objectives

The primary objective was to evaluate reasons for readmission and identify preventable and nonpreventable readmissions. Secondary objectives included identifying TOR, LOS, 90-day readmission rate, 90-day reoperation rate, and mortality rate before and after hip fracture protocol implementation.

Demographic variables extracted included patient age, sex, race, body mass index (BMI), and the American Society of Anesthesiologists (ASA) classification. Procedure Type (hemiarthroplasty, total hip replacement, or open reduction internal fixation (ORIF) was also recorded.

Statistical analysis

Patient demographic and outcome variables were recorded, with counts and percentages utilized for categorical variables and mean plus standard deviation (SD) utilized for continuous variables. For univariate analysis, a Chi-squared test was performed to compare differences between the two cohorts for categorical variables, and a Student's t-test was performed for continuous variables.

A multivariate logistic regression analysis was performed on the five outcome variables of interest to identify the odds ratio (OR), 95% confidence interval (CI), and p-value between patients treated after 2016 and those treated before 2016. The analysis controlled for patient demographic factors (age, sex, race, and BMI), as well as clinical factors (LOS and procedure type). A forest plot of the odds ratios was plotted using Excel (Microsoft, Inc., Redmond, WA). All analyses were performed using IBM Corp. Released 2021. IBM SPSS Statistics for Windows, Version 28.0. Armonk, NY: IBM Corp. A significance level of p < 0.05 was set for both univariate and multivariate analyses.

This study was reviewed by the Yale University Institutional Review Board (IRB) and received an IRB exemption (ID: 2000031610).

## Results

This retrospective cohort study identified 163 patients who were treated for a fragility hip fracture between January 2014 and January 2016 (control group). Of those patients, 91 (55.8%) underwent hemiarthroplasty, 12 (7.4%) underwent THA, and 60 (36.8%) underwent cephellomedullary nail/ORIF. In the intervention group, 238 patients were identified who were treated for a fragility hip fracture between February 2016 and February 2018. Of those patients, 145 (60.9%) underwent hemiarthroplasty, 33 (13.9%) underwent THA, and 60 (25.2%) underwent cephellomedullary nail/ORIF. No statistically significant difference was identified between the control and intervention groups with regard to age, sex, race, BMI, or ASA score (Table 1).

**Table 1:** Patient demographics Patient demographics for fragility hip fracture patients before and after 2016. ASA: American Society of Anesthesiologists Score, BMI: Body Mass Index, ORIF: Open Reduction Internal Fixation, SD: Standard Deviation Empty cells, not applicable. Analysis performed for each aggregate categorical and continuous variable

	2014-2016		2016-2018		
	N (%)	Mean (SD)	N (%)	Mean (SD)	p-value
Total	163		238		
Age		81 (10.0)		81 (10.0)	0.908
Sex					0.752
Female	110 (67.5%)		157 (66.0%)		
Male	53 (32.5%)		81 (34.0%)		
Race					0.527
White	146 (89.6%)		218 (91.6%)		
Black	10 (6.1%)		7 (2.9%)		
Hispanic	5 (3.1%)		7 (2.9%)		
Asian	1 (0.6%)		3 (1.3%)		
Other	1 (0.6%)		3 (1.3%)		
BMI					0.427
ASA					0.081
1	0 (0.0%)		5 (2.1%)		
2	24 (14.7%)		51 (21.4%)		
3	114 (69.9%)		151 (63.4%)		
4	25 (15.3%)		31 (13.0%)		
Procedure	-		-		0.015*
Hemi	91 (55.8%)		145 (60.9%)		
Total	12 (7.4%)		33 (13.9%)		
ORIF	60 (36.8%)		60 (25.2%)		

TOR averaged 33.2 hours for the control group and 33.3 hours for the intervention group. However, 52.5% of patients in the intervention group and 42.9% of patients in the control group underwent surgery within 24 hours of admission. LOS was noted to be shorter by 1.1 days for the intervention group (p = 0.010, Table 2). 

**Table 2:** Univariate outcomes Univariate outcomes comparing fragility hip fracture patients before and after 2016. AMS: Altered Mental Status, CVA: Cardiovascular Accident, GI: Gastrointestinal, LOS: Length of Stay, OR: Operating Room, SD: Standard Deviation, SSI: Surgical Site Infection, UTI: Urinary Tract Infection, VTE: Venous Thromboembolism *p<0.05

	2014-2016		2016-2018		
	N (%)	Mean (SD)	N (%)	Mean (SD)	p-value
Time to OR (Hours)		33.2 (26.7)		33.3 (42.1)	0.983
OR Time					0.040*
<24 hours	70 (42.9%)		125 (52.5%)		
24-36 hours	40 (24.5%)		61 (25.6%)		
>36 hours	53 (32.5%)		51 (21.4%)		
Reoperations	6 (3.7%)		9 (3.8%)		0.958
LOS (Mean, SD)		6.3 (5.2)		5.2 (3.4)	0.010*
90 Day Readmit	42 (25.8%)		78 (32.8%)		0.112
Medical Total	33 (20.2%)		68 (28.6%)		0.059
Anemia	1 (0.6%)		3 (1.3%)		
AMS	4 (2.5%)		1 (0.4%)		
Cardiac	3 (1.8%)		10 (4.2%)		
CVA	0 (0.0%)		2 (0.8%)		
Endocrine	0 (0.0%)		4 (1.7%)		
Fall (Unrelated to site)	0 (0.0%)		7 (2.9%)		
GI	7 (4.3%)		5 (2.1%)		
Hepatic	0 (0.0%)		1 (0.4%)		
Pulmonary	13 (8.0%)		13 (5.5%)		
Renal	4 (2.5%)		6 (2.5%)		
Sepsis	0 (0.0%)		2 (0.8%)		
UTI	1 (0.6%)		4 (1.7%)		
VTE	0 (0.0%)		3 (1.3%)		
Other	0 (0.0%)		7 (2.9%)		
Surgical Total	9 (5.5%)		10 (4.2%)		0.541
Dislocation	2 (1.2%)		3 (1.3%)		
Periprosthetic fracture	2 (1.2%)		1 (0.4%)		
Hematoma	0 (0.0%)		1 (0.4%)		
Nonunion	0 (0.0%)		1 (0.4%)		
SSI	4 (2.5%)		4 (1.7%)		
Vascular	1 (0.6%)		0 (0.0%)		
90 Day Mortality	21 (12.9%)		16 (6.7%)		0.036*
1 Year Mortality	38 (23.3%)		35 (14.7%)		0.028*
All Mortality	86 (52.8%)		67 (28.2%)		<0.001*

**Figure 1:**
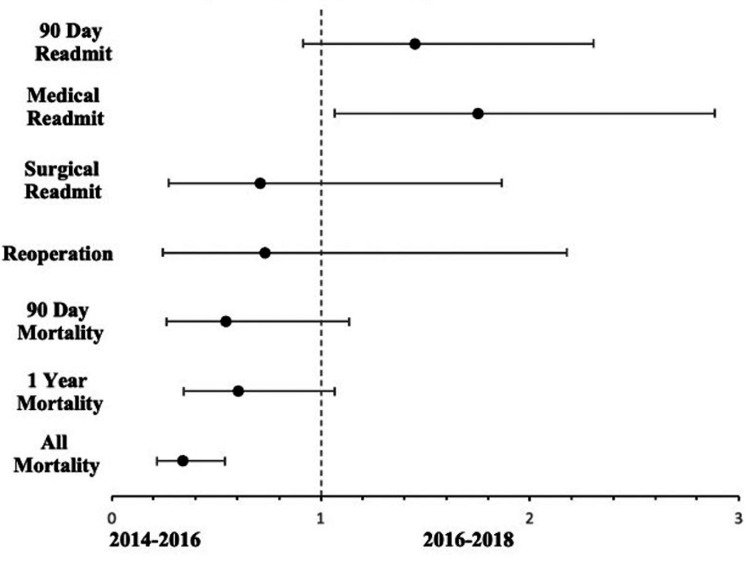
Forest plot comparing the odds ratios of the 2016-2018 cohort with 2014-2016 as the referent cohort Odds of 90-day readmissions, medical readmissions, surgical readmissions, reoperations, 90-day, one year, and all mortality were compared, controlling for age, sex, race, BMI, LOS, and procedure type in the multivariate analysis.

No statistically significant difference was seen in terms of 90-day overall readmission rates between the two groups on both univariate and multivariate analysis after controlling for age, sex, race, BMI, ASA, LOS, and procedure (p = 0.112 and 0.113, respectively, Table 3). 79% of readmissions in the control group and 87% of readmissions in the intervention group were secondary to a medical reason. On multivariate analysis, patients in the intervention group were found to have higher odds of being readmitted for a medical reason compared with patients in the control group (p = 0.028, Table 3 and Figure 1). A pulmonary etiology was the most common medical reason for readmission but was less prevalent in the intervention group (19%) compared with the control group (39%). 15% of patients in the intervention group (n = 10) vs. 9% in the control group (n = 3) were readmitted for a cardiac etiology. Of the patients readmitted for a GI etiology, 43% in the control group (n = 3) and 40% (n = 2) in the intervention group were due to a GI bleed. Seven patients were readmitted for other medical reasons in the intervention group, which included chronic pain due to spinal stenosis, failure to thrive, intractable back pain, weakness, subarachnoid hemorrhage, seizure, and bipolar disorder. Additional medical reasons for readmission included anemia, altered mental status (AMS), cerebrovascular accidents (CVA), endocrine, fall with an injury unrelated to the surgical site, hepatic, renal, sepsis, urinary tract infection (UTI), and venous thromboembolism (VTE) (Table 2).

No statistically significant differences were seen between the groups with regard to surgical readmissions (p = 0.158). Reasons for readmission included dislocation, periprosthetic fractures, hematoma of the surgical site, nonunion, and surgical site infection (SSI). One patient in the control group was readmitted for a pseudoaneurysm near the surgical site and underwent a thrombin injection by interventional radiology without complications.

No difference was seen in terms of the reoperation rate between the two groups (p =.958). Reoperation reasons for the control group included recurrent dislocation (n = 2), contralateral hip fracture (n = 2), and surgical site infection (n = 2). Reoperation reasons for the intervention group included dislocation (n = 2), periprosthetic fracture (n = 1), surgical site infection (n = 4), wound hematoma (n = 1), and nonunion (n = 1).

Overall, no patients were readmitted to the hospital for social issues. 90-day, one-year, and all mortality on univariate analysis were significantly lower in the intervention group vs. the control group (Table 2). However, with multivariate analysis, statistical significance was only achieved for all mortality (Table 3 and Figure 1).

**Table 3:** Multivariate logistic regression Multivariate logistic regression comparing fragility hip fracture patients before and after 2016 (referent: 2014-2016) while controlling for age, sex, race, BMI, LOS, and procedure. *p<0.05

	OR	95%	CI	p-value
90 Day Readmit	1.452	0.915	2.304	0.113
Medical Readmit	1.753	1.064	2.887	0.028*
Surgical Readmit	0.712	0.271	1.865	0.489
Reoperations	0.732	0.246	2.176	0.575
90 Day Mortality	0.545	0.262	1.133	0.104
One-Year Mortality	0.604	0.343	1.065	0.082
All Mortality	0.341	0.215	0.542	<0.001*

## Discussion

This study investigated the outcomes of geriatric patients who underwent surgery for a hip fracture either before or after the implementation of a comprehensive and standardized hip fracture protocol. The findings from this study validate our hypothesis. Since the implementation of the hip fracture program, a greater percentage of patients underwent surgery within 24 hours and had a reduced LOS compared with the control group before protocol implementation. The hip fracture protocol was also associated with reduced 90-day and one-year mortality, which was statistically significant on univariate analysis. These findings are consistent with other studies investigating hip fracture programs and protocols [6,9].

However, despite the incorporation of a comprehensive preoperative, intraoperative, and postoperative hip fracture protocol, 90-day readmissions were not significantly reduced in our study’s intervention group. Medical problems were the main reasons for readmission for both groups, but significantly higher in the intervention group on multivariate analysis. On the other hand, surgical readmissions were insignificantly lower in the intervention group. These findings suggest we may be minimizing surgical-related readmissions, but it is unclear why medical readmissions have increased, especially with improvements in our hip fracture protocol. Additional efforts are needed to address medical-related readmissions in this patient population.

In a study by Stahl et al., pneumonia and postoperative delirium were identified as having the largest impact on readmissions as well as LOS and mortality after a fragility hip fracture [10]. Kates et al. demonstrated that pneumonia followed by congestive heart failure and atrial fibrillation were the most common medical reasons for readmission after hip fracture surgery [11]. In our intervention group, only one patient (0.01%) was readmitted for AMS vs. four patients (12%) in the control group. Similar to previous studies, pulmonary etiologies for readmission were the most prevalent medical reason for readmission for both groups but were less common in the intervention group compared with the control group. These findings are possibly explained by the routine delirium assessments, catheter protocol, oral care, and early mobilization incorporated into our hip fracture protocol [12-14].

Readmissions related to postoperative bleeding (i.e., anemia, GI bleeding, subarachnoid hemorrhage, and surgical site hematomas) can be related to DVT prophylaxis but may be difficult to prevent. The risk of bleeding must be weighed against the risk of developing a DVT and, subsequently, a pulmonary embolism (PE). It would be unreasonable, unless contraindicated due to high-risk bleeding, to not give hip fracture patients DVT prophylaxis. These patients are at particularly high risk for DVT/PE, especially when surgery is delayed beyond 48 hours [15]. From the two cohorts, not one patient was readmitted for a DVT or PE.

Ongoing efforts should be taken to help minimize readmissions, which not only negatively impact patients but are also associated with increased healthcare costs and LOS. Kates et al. demonstrated a longer readmission LOS (8.7 ± 18.8 days) compared with patients’ initial LOS (4.6 ± 2.3 days) after hip fracture surgery [11]. Checketts et al. noted the importance of identifying patients who had previous inpatient or emergency visits within the year before their hip fracture surgery and suggested special efforts be taken to minimize readmissions in this population [16]. Potential areas of improvement and additional study may include closer internal medicine oversight and primary care follow-up after discharge. 

In addition to readmission rates, variables such as LOS, complications, reoperation rate, and mortality rate are commonly used as a metric for hospital performance and help determine the effectiveness of a hip fracture program [6]. However, as demonstrated in our study, readmission reasons can be variable and difficult to prevent even with a comprehensive hip fracture protocol in place. In addition, some payment models often penalize hospitals for readmissions [17,18]. This may be unjust, especially for those taking care of patients with multiple medical comorbidities who are at high risk for readmission and have non-modifiable risk factors. Thus, penalizing hospitals for readmissions may be unreasonable, and various factors need to be considered when developing alternative payment models.

A strength of this study is that it was based on a comprehensive hip fracture program that standardized preoperative, intraoperative, and postoperative care and was followed by all surgeons, both faculty and community. In addition, we comment on specific reasons for readmission that previous studies routinely fail to mention. Another strength is the multivariate analysis used, which controlled for potential confounding variables.

Some limitations of our study include its retrospective design as well as its relatively small sample size. A more powerful study could possibly achieve statistical significance on multivariate analysis for 90-day and one-year mortality and may also help identify differences with regard to specific readmission reasons between the groups.

## Conclusions

A comprehensive hip fracture protocol standardizing preoperative, intraoperative, and postoperative care can lead to reduced LOS, faster TOR, reduced mortality, and possibly reduced surgical-related readmissions. However, overall readmission rates may not be an adequate marker for hip fracture protocol effectiveness, as many of the readmissions were secondary to medical problems that may be unavoidable. Some readmission penalties may be unreasonable, and various factors need to be considered for alternative payment models. Ongoing efforts should be taken to identify ways to reduce readmissions without penalizing hospitals, especially those with comprehensive hip fracture programs. Potential areas of improvement and additional study may include closer internal medicine oversight and primary care follow-up after discharge. 
